# Association between cardiovascular health and pelvic inflammatory disease: Analyses of the NHANES 2015 to 2018

**DOI:** 10.1097/MD.0000000000038981

**Published:** 2024-07-19

**Authors:** Yang Yang, Kewei Chen, Huaifang Li, Xiaowen Tong

**Affiliations:** a Department of Obstetrics and Gynecology, Tongji Hospital, School of Medicine, Tongji University, Shanghai, China.

**Keywords:** cardiovascular health, life essential 8, NHANES, pelvic inflammatory disease

## Abstract

While the link between female reproductive function and cardiovascular health (CVH) is well-established, the association between pelvic inflammatory disease (PID) and CVH remains largely unexplored. This study, therefore, sets out to fill this gap in knowledge by investigating the potential relationship between PID and CVH. To ensure the reliability and validity of our findings, data for this cross-sectional study were meticulously collected from the 2015–2018 National Health and Nutrition Examination Survey (NHANES). After applying stringent exclusion criteria, a total of 2442 women were included in the study. The Life Essential 8 (LE8) scoring system, a robust tool developed by the American Heart Association (AHA), was employed to assess the CVH. Logistic regression with multiple variables and smooth curve fitting were utilized to analyze the association. Subgroup and interaction analyses were performed to determine the strength of this association across different demographic groups. The study included 2442 women, with an average CVH score of 66.29 ± 16.27. After accounting for all covariates, each unit increase in CVH score was associated with 2% lower odds of PID prevalence (OR = 0.98, 95% CI: 0.97–0.99). Notably, participants with high CVH had a striking 71% lower odds of PID prevalence compared to those with low CVH. Stratified analyses further revealed a consistent inverse association between CVH score and PID across various subgroups, underscoring the robustness of our findings. The research has uncovered a significant inverse association between CVH and PID. This suggests that improving the CVH level could be a promising strategy for reducing the odds of PID.

## 1. Introduction

Pelvic inflammatory disease (PID) is a condition in women that results from a multi-microbial infection in the upper vaginal tract. It is especially common in women of reproductive age, making it a significant health concern for this population.^[1]^ The global prevalence of PID is roughly 4% to 12%.^[2]^ Without effective treatment, severe cases of PID can develop into tubo-ovarian abscesses.^[3]^ Other sequelae, excluding infertility and ectopic pregnancy, are also closely related to the development of gynecological cancers.^[4,5]^ In addition, recurrent episodes of PID can burden society and the healthcare system.^[6]^ Thus, investigating the risk factors linked to PID is crucial for offering early interventions.

In 2010, the American Heart Association (AHA) introduced Life Simple 7 (LS7), a set of health guidelines created to act as an assessment tool cardiovascular health (CVH).^[7]^ Building on LS7, the AHA revised the Life Essential 8 (LE8) with the goal of improving overall public health. Notably, LE8 provides a more precise stratification of CVH than LS7, combining advanced grading techniques and considerations for sleep quality.^[8]^ LE8 has been used as a tool for CVH assessment in many diseases. Examples include cardiovascular disease,^[9]^ cognitive dysfunction,^[10]^ depression,^[11]^ and periodontitis.^[12]^

However, there has been limited exploration into the potential association between CVH and PID. Therefore, we undertook a study utilizing data from the 2015 to 2018 National Health and Nutrition Examination Survey (NHANES) to investigate this relationship. Our results provide valuable insights that could inform PID prevention strategies in clinical settings.

## 2. Methods

### 2.1. Study population and design

**Figure 1.**
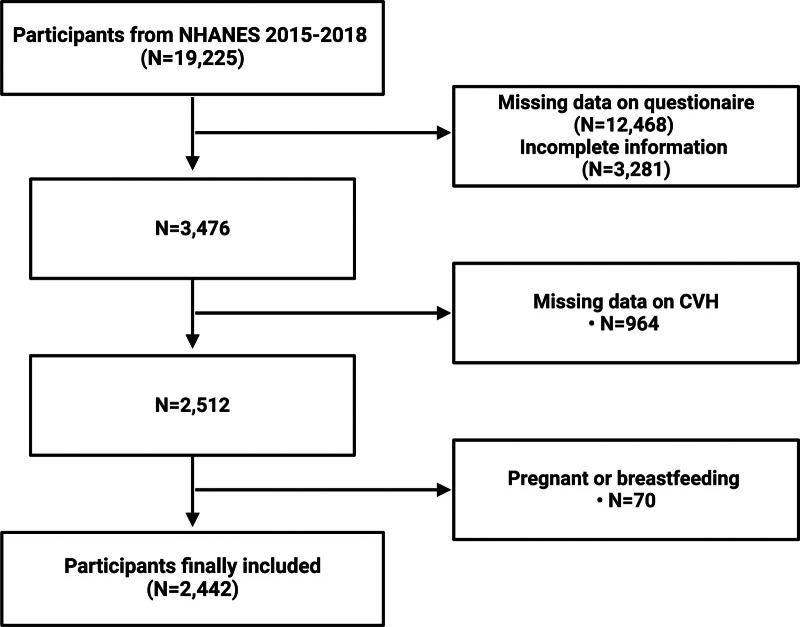
Flow chart of participants selection. NHANES = National Health and Nutrition Examination Survey.

The data utilized in this study were sourced from NHANES, a nationally representative cross-sectional survey exploring nutrition and health status within the United States. Every participant in NHANES provided written informed consent, and the National Center approved all research procedures for the Health Statistics Research Ethics Review Board. The data from the 2015 to 2018 NHANES cycle were included in the scope of the current investigation. PID only affects the female upper vaginal tract. Hence, only female participants were chosen. Initially, participants were interviewed at their residences using demographic questionnaires for the individual and the family. This was followed by either an additional interview or a health assessment. In the 2 cycles under consideration, a combined total of 19,225 individuals participated. Following the removal of individuals with missing questionnaire data (N = 12,468), incomplete information (N = 3281), absent CVH data (N = 964), and pregnant or breastfeeding status (N = 70), the analysis included 2442 participants (see Fig. 1).

### 2.2. Evaluation of PID

The “RHQ078” questionnaire asked: “Have you ever been treated for an infection in the fallopian tubes, uterus, or ovaries, also called a pelvic infection, pelvic inflammatory disease, or PID?” was used to diagnose PID.^[13]^ The PID is determined based on a yes or no answer. Individuals who declined to respond, provided an ambiguous answer, or did not fill out a PID diagnosis survey were omitted from the analysis.

### 2.3. CVH evaluation

LE8 comprises 8 components: body mass index (BMI), blood glucose, blood pressure, diet, blood lipids, nicotine exposure, physical activity, and sleep health.^[8]^ CVH is assessed through the use of the LE8 tool. The algorithm for LE8 was previously reported in the literature.^[14]^ The diet score is rooted in the Dietary Approaches to Stop Hypertension dietary measure. The laboratory provided data on lipids, blood glucose levels, and weight. Information on physical activity, exposure to nicotine, duration of sleep, and status of diabetes were all gathered through the use of a questionnaire. The scores for the 8 factors were weighted to give scores ranging from 0–100. According to guidelines from the AHA, individuals were categorized into 3 groups based on their LE8 scores: low (<50), medium (50–79), and high CVH (more than 79).^[8]^

### 2.4. Covariates

Our analysis included a variety of previously recognized risk variables for CVH or PID, including age, marital status, race/ethnicity, education levels, and the ratio of family income to poverty (PIR). Physical exams were performed to detect characteristics such as BMI.

### 2.5. Statistical analysis

All statistical analysis followed the Centers for Disease Control and Prevention recommendations, including appropriate NHANES sampling and accounting for a complicated multistage cluster survey. Continuous variables were presented as means with standard deviation (SD), and categorical variables were reported as frequencies with percentages. Multivariable logistic regression analysis was employed to assess connections between PID and CVH. Model 1 was unadjusted for variables; Model 2 adjusted for age and race; Model 3 adjusted for age, race, marital status, education level, and PIR. There were no extra adjustments for clinical factors such as diabetes or hypertension. Because the measures were previously taken into consideration when estimating the LE8 scores. To examine potential relationships between CVH and PID, we utilized a smooth curve fitting. Subgroup analyses, including age, race/ethnicity, education level, and PIR, were conducted to explore potential effect modifiers. Interaction terms were used to assess subgroup heterogeneity. This study utilized 2-sided statistical testing, with *P* < .05 showing statistical significance. Data analysis was performed using R (version 4.1.1) or Empowerstats (version 4.1).

## 3. Results

### 3.1. Baseline characteristics

The baseline characteristics of the demographics were shown for different CVH subgroups in Table 1. The present analysis includes 2442 participants. The average age was 40.36 ± 11.46 years. The mean (SD) for the CVH score was 66.29 ± 16.27. There were 394 (16.13%) participants in the low CVH category, 1488 (60.93%) in the moderate CVH category, and 560 (22.93%) in the high CVH category. In terms of age, ethnicity, marital status, level of education, and PIR, there was a significant difference in the scores of CVH among the 3 groups (*P* < .001). The average prevalence of PID was 6.31% overall, with rates of 11.42%, 6.52%, and 2.14% in the low, moderate, and high CVH categories, respectively. As CVH scores increased, the prevalence of PID decreased. Initial assessments indicated that participants classified with high CVH tended to be younger and of White ethnicity. Moreover, they were more inclined to possess higher levels of education and reported substantial household earnings.

**Table 1:** Baseline characteristics of participants with different CVH levels estimated from LE8 score.

Characteristics	Total	Low (LE8 < 50)	Moderate (50 ≤ LE8 < 80)	High (LE8 ≥ 80)	*P* value
No. of participants in sample	2442	394	1488	560	
Age, yr (SD)	40.36 ± 11.46	45.95 ± 9.77	40.58 ± 11.33	35.84 ± 11.05	<.001
PIR	2.50 ± 1.63	2.05 ± 1.48	2.55 ± 1.63	3.03 ± 1.71	
Marital status, n(%)					<.001
Married	1121 (45.90%)	156 (39.59%)	681 (45.77%)	284 (50.71%)	
Widowed	56 (2.29%)	18 (4.57%)	35 (2.35%)	3 (0.54%)	
Divorced	275 (11.26%)	70 (17.77%)	160 (10.75%)	45 (8.04%)	
Separated	111 (4.55%)	26 (6.60%)	78 (5.24%)	7 (1.25%)	
Never married	586 (24.00%)	84 (21.32%)	342 (22.98%)	160 (28.57%)	
Living with partner	293 (12.00%)	40 (10.15%)	192 (12.90%)	61 (10.89%)	
Race/ethnicity, n(%)					<.001
Non-Hispanic White	816 (33.42%)	142 (36.04%)	455 (30.58%)	219 (39.11%)	
Non-Hispanic Black	547 (22.40%)	119 (30.20%)	364 (24.46%)	64 (11.43%)	
Mexican American	414 (16.95%)	68 (17.26%)	268 (18.01%)	78 (13.93%)	
Other Hispanic	408 (16.71%)	36 (9.14%)	240 (16.13%)	132 (23.57%)	
Others	257 (10.52%)	29 (7.36%)	161 (10.82%)	67 (11.96%)	
Education level, n(%)					<.001
Less than high school	357 (14.62%)	97 (24.62%)	226 (15.19%)	34 (6.07%)	
High school	513 (21.01%)	111 (28.17%)	318 (21.37%)	84 (15.00%)	
More than high school	1572 (64.37%)	186 (47.21%)	944 (63.44%)	442 (78.93%)	
PID, n(%)					<.001
Yes	154 (6.31%)	45 (11.42%)	97 (6.52%)	12 (2.14%)	
No	2288 (93.69%)	349 (88.58%)	1391 (93.48%)	548 (97.86%)	
AHA LE8 score (SD)					
Total CVH score	66.29 ± 16.27	41.30 ± 6.65	64.78 ± 8.30	87.87 ± 5.42	<.001
Mean DASH diet score	41.73 ± 32.55	24.49 ± 26.84	37.98 ± 30.74	63.79 ± 29.54	<.001
Mean physical activity score	47.36 ± 47.25	7.84 ± 23.59	41.58 ± 46.15	90.52 ± 25.21	<.001
Mean tobacco/nicotine exposure score	72.14 ± 37.75	42.88 ± 40.75	72.55 ± 37.23	91.63 ± 19.62	<.001
Mean sleep health score	83.67 ± 24.20	70.10 ± 29.14	83.85 ± 23.84	92.75 ± 15.35	<.001
Mean body mass index score	54.20 ± 37.02	25.23 ± 28.71	50.75 ± 35.24	83.73 ± 24.57	<.001
Mean blood lipid score	72.81 ± 30.19	50.20 ± 31.78	72.22 ± 28.84	90.29 ± 19.76	<.001
Mean blood glucose score	84.37 ± 25.11	62.06 ± 30.91	85.17 ± 23.47	97.93 ± 8.87	<.001
Mean blood pressure score	74.03 ± 30.01	47.60 ± 30.30	74.16 ± 28.72	92.29 ± 16.39	<.001

Mean (SD) for continuous variables: the *P* value was calculated by the linear regression model.

Percentages for categorical variables: the *P* value was calculated by the chi-square test.

Cardiovascular health (CVH) is categorized into 3 grades, low: LE8 score < 50, medium: 50 ≤ LE8 score < 80, high: LE8 score ≥ 80.

AHA = American Heart Association, CVH = cardiovascular health, DASH = dietary approaches to stop hypertension, LE8 = life’s essential 8, PID = pelvic inflammatory disease, PIR = the ration of family income to poverty.

### 3.2. Association between CVH and PID

Table 2 illustrates the relationship between the total CVH score and PID. In Model 1, which did not adjust for any variables, the data indicated that the OR for the CVH score was 0.97 (95% CI: 0.96–0.98), suggesting a significant link with the odds of PID. When age and ethnicity variables were controlled, the negative association remained statistically significant in Model 2 (OR 0.97, 95%CI: 0.96–0.98). After accounting for all covariates, each increment in the CVH score was associated with a 2% reduction in the odds of PID prevalence (OR 0.98, 95%CI: 0.97–0.99). When participants achieved ideal scores on the CVH score, there was a notable reduction in the likelihood of PID when these scores were analyzed as a categorical variable. This trend was further supported by the significantly lower OR for PID among those with ideal scores compared to those with poor scores. These results remained consistent even after adjusting for covariates in Model 2. Additionally, in Model 3, where all covariates were controlled for, it was observed that the subgroup with high CVH scores had 71% lower odds of PID prevalence (OR 0.29, 95% CI: 0.14–0.59) compared to the subgroup with low CVH scores. Therefore, the analysis indicates a strong association between CVH and the likelihood of PID.

**Table 2:** The association between the life essential 8 cardiovascular health (CVH) score and pelvic inflammatory disease (PID).

	Model 1 [OR (95% CI) *P*]	Model 2 [OR (95% CI) *P*]	Model 3 [OR (95% CI) *P*]
Total CVH score	0.97 (0.96, 0.98) <.001	0.97 (0.96, 0.98) <.001	0.98 (0.97, 0.99) .001
CVH categories			
Low (LE8 < 50)	1	1	1
Moderate (50 ≤ LE8 < 80)	0.54 (0.37, 0.78) .001	0.60 (0.41, 0.88) .009	0.69 (0.47, 1.04) .074
High (LE8 ≥ 80)	0.17 (0.09, 0.33) <.001	0.21 (0.11, 0.41) <.001	0.29 (0.14, 0.59) < .001
*P* for trend	<.01	<.01	<.01

Model 1 was unadjusted for covariates; Model 2 enhanced Model 1 by including age and ethnicity; and Model 3 further augmented Model 2 by integrating marital status, education level, and family income-to-poverty ratio.

CVH = cardiovascular health, PID = pelvic inflammatory disease.

**Figure 2.**
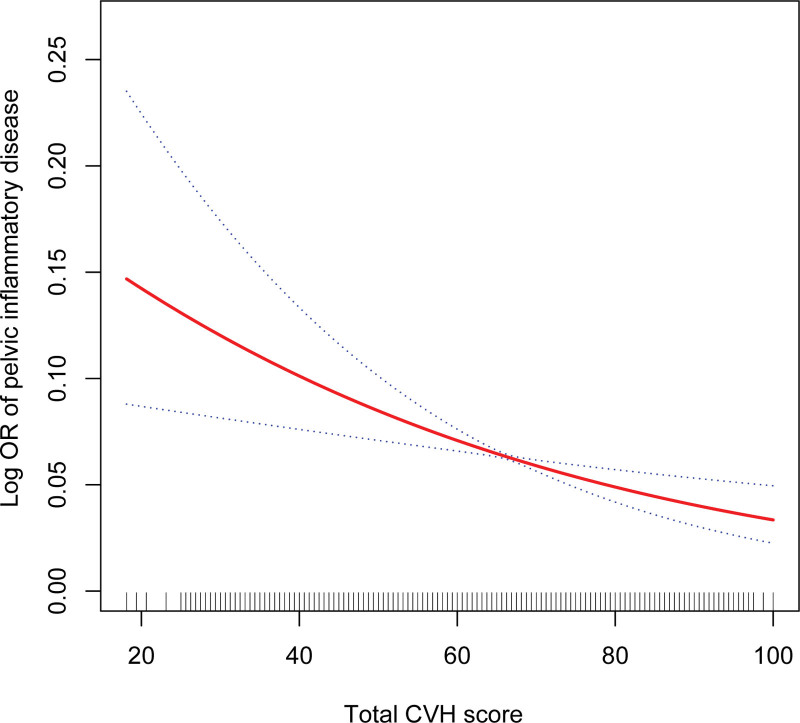
Smooth curve fittings.

Smooth curve fitting further demonstrated the negative relationship between CVH score and PID (Fig. 2).

### 3.3. Subgroup analysis

Utilizing subgroup analysis, the study evaluated the association between CVH scores and PID (as shown in Table 3). This link remains steady across various groups, including age, ethnicity, level of education, and PIR (with *P* values exceeding .05 for all interactions). These results reveal consistent inverse associations across demographics, suggesting that there may be broader applicability across populations.

**Table 3:** Subgroup analysis of the association between CVH and pelvic inflammatory disease.

Subgroup	PID [OR (95%CI)]	*P* for interaction
Age		.102
<40 yr	0.97 (0.95, 0.99)	
≥40 yr	0.99 (0.97, 1.00)	
Race		.259
Non-Hispanic White	0.98 (0.96, 1.00)	
Non-Hispanic Black	1.00 (0.97, 1.02)	
Mexican American	0.99 (0.95, 1.02)	
Other Hispanic	0.95 (0.92, 0.99)	
Others	0.99 (0.95, 1.02)	
Education level		.502
Less than high school	1.00 (0.97, 1.03)	
High school	0.99 (0.97, 1.01)	
More than high school	0.98 (0.96, 0.99)	
PIR		.622
<1.3	0.97 (0.96, 0.99)	
1.3–3.5	0.98 (0.97, 1.00)	
>3.5	0.99 (0.96, 1.01)	

Age, marital status, race, education level, and PIR.

CVH = cardiovascular health, PID = pelvic inflammatory disease, PIR = the ratio of income to poverty.

## 4. Discussion

In a cross-sectional study involving 2442 participants, we examined the association between CVH and PID, and our findings revealed an inverse association. This connection was found in subgroups stratified by age, race, education level, and PIR, suggesting robustness across various demographic characteristics. This study is among the first to demonstrate a potential link between CVH scores and PID, offering a novel perspective for further investigation into the role of comprehensive health behaviors and factors in PID prevention.

This is the first study on the relationship between CVH and PID. Previous studies have generally associated cardiovascular disease with female reproductive complications,^[15]^ polycystic ovary syndrome,^[16]^ and endometriosis.^[17]^ There are very few studies on PID and cardiovascular disease. Previous studies have also focused on the association of PID with specific cardiovascular diseases.^[18,19]^ For instance, research from Taiwan indicated that patients with PID had a higher likelihood of myocardial infarction compared to controls.^[20]^ However, understanding the association between CVD and PID requires consideration of the shared pathogenesis and common risk factors between these conditions. LE8 includes common risk variables associated with PID and cardiovascular. In terms of health factors, for example, a recent retrospective matched cohort research from the United Kingdom found that PID can lead to an increased incidence of hypertension and diabetes, both of which are also high-risk factors for cardiovascular disease.^[21]^ A retrospective study from Japan found that obesity prolongs hospitalization for PID.^[6]^ Obesity has been shown to impair immunological response by generating pro-inflammatory substances in adipose tissue and affecting T-cell activity.^[22]^ Similarly, investigations have linked overweight status, as well as obesity classes I, II, and III, to heightened risks of endometritis subsequent to vaginal, non-instrumental deliveries.^[23]^

Regarding health behavior, Juanjuan Ma et al^[24]^ elucidated a noteworthy positive correlation between dietary inflammatory index (DII) scores and susceptibility to PID. This study emphasizes the potential of anti-inflammatory diet therapy as a new therapeutic strategy for PID. The DII index is used to verify the anti-inflammatory level of the diet. In addition, in a study on dietary minerals and PID, copper intake in relation to PID was influenced by the interaction of age and BMI.^[13]^ Copper has also been shown to have anti-inflammatory effects in many diseases.^[25]^ Another study showed that smoking prolonged hospitalization for PID.^[26]^ In addition, PID was found to be associated with sleep, a factor added in LE8. PID has been linked to an elevated risk of sleep disturbances, consequently compromising the overall quality of life.^[27]^

The mechanisms governing the inverse relationship between CVH and the likelihood of PID are complex and extensive. One possible mechanism entails the direct infiltration of the arterial vasculature.^[28]^ Chlamydia trachomatis was responsible for most PID diagnoses.^[29]^ In previous studies, Chlamydia trachomatis cardiovascular disease has been confirmed in case reports or experimental studies,^[30,31]^ and a recent study detected Chlamydia trachomatis in the heart valves of patients with coronary artery disease. It was shown that Chlamydia trachomatis can invade arterial walls and cause inflammation, leading to cardiovascular disease.^[32]^ Numerous investigations have detected the presence of Chlamydia trachomatis within cardiovascular tissues, suggesting its potential involvement in cardiovascular disease pathogenesis through localized effects.^[32,33]^ This direct infiltration and resultant inflammation could link PID to CVH. The second mechanism may be a systemic inflammatory response. Inflammatory response due to PID activates immune regulation. This immune activation involves the release of pro-inflammatory cytokines, chemokines, and other immune mediators to combat bacterial infection in the pelvic organs.^[34]^ These inflammatory molecules can also exert systemic effects, impacting CVH. Pro-inflammatory factors have been implicated in the development of several cardiovascular diseases.^[35]^ These cytokines include interleukin-6, tumor necrosis factor (TNF) alpha, and the interleukin-1 family. Chronic inflammation is a known risk factor for CVD,^[36]^ and the persistent inflammatory state induced by PID could, therefore, contribute to a deterioration in CVH. Additionally, lifestyle factors that improve CVH, such as a healthy diet, regular exercise, and smoking cessation, may also reduce the prevalence of PID. These behaviors are associated with lower systemic inflammation and improved immune function,^[37,38]^ which can protect against infections that lead to PID.

Our research is the first to investigate the relationship between CVH and PID. An intensive association between CVH and PID was identified. However, there were several limitations to this research. Firstly, despite our efforts to control for various potential confounding factors, the cross-sectional nature of our inquiry precludes definitive conclusions regarding the causality between CVH and PID. Further investigation into the longitudinal and causal relationship between CVH and PID is imperative. Secondly, utilizing a PID questionnaire for diagnostic purposes unavoidably introduced a degree of selection bias. Thirdly, LE8 is assessed through surveys and is subject to some errors. Fourthly, the influence of nonrandom missing data on outcomes should not be underestimated. Finally, applying our results to younger demographics or individuals from varied geographical areas requires further investigation to confirm generalizability.

## 5. Conclusion

This study demonstrates an inverse association between CVH and PID. These findings emphasize the significance of maintaining CVH to decrease the incidence of PID. The causal relationship between CVH and PID needs to be further investigated in future studies, and the specific mechanisms linking the 2 need to be elucidated.

## Acknowledgments

We would thank Ms Min Liu for her guidance on the statistics.

## Author contributions

**Conceptualization:** Xiaowen Tong.

**Data curation:** Yang Yang, Kewei Chen.

**Formal analysis:** Yang Yang, Kewei Chen.

**Investigation:** Yang Yang, Kewei Chen.

**Methodology:** Xiaowen Tong, Yang Yang, Huaifang Li.

**Project administration:** Xiaowen Tong, Yang Yang, Huaifang Li.

**Supervision:** Xiaowen Tong, Yang Yang, Huaifang Li.

**Writing – original draft:** Xiaowen Tong, Yang Yang.

**Writing – review & editing:** Xiaowen Tong, Yang Yang.
